# Two-year trajectory of functional recovery and quality of life in post-intensive care syndrome: a multicenter prospective observational study on mechanically ventilated patients with coronavirus disease-19

**DOI:** 10.1186/s40560-025-00777-z

**Published:** 2025-02-06

**Authors:** Junji Hatakeyama, Kensuke Nakamura, Shigeaki Inoue, Keibun Liu, Kazuma Yamakawa, Takeshi Nishida, Shinichiro Ohshimo, Satoru Hashimoto, Naoki Kanda, Shotaro Aso, Shinya Suganuma, Shuhei Maruyama, Yoshitaka Ogata, Akira Takasu, Daisuke Kawakami, Hiroaki Shimizu, Katsura Hayakawa, Takeshi Yoshida, Taku Oshima, Tatsuya Fuchigami, Hironori Yawata, Kyoji Oe, Akira Kawauchi, Hidehiro Yamagata, Masahiro Harada, Yuichi Sato, Tomoyuki Nakamura, Kei Sugiki, Takahiro Hakozaki, Satoru Beppu, Masaki Anraku, Noboru Kato, Tomomi Iwashita, Hiroshi Kamijo, Yuichiro Kitagawa, Michio Nagashima, Hirona Nishimaki, Kentaro Tokuda, Osamu Nishida

**Affiliations:** 1 Department of Emergency and Critical Care Medicine, National Hospital Organization Tokyo Medical Center, 2-5-1 Higashigaoka, Meguro-ku, Tokyo, 152-8902 Japan; 2 Department of Emergency and Critical Care Medicine, Osaka Medical and Pharmaceutical University, 2-7 Daigaku-machi, Takatsuki, Osaka 569-8686 Japan; 3 Department of Critical Care Medicine, Yokohama City University School of Medicine, 3-9 Fukuura, Kanazawa-ku, Yokohama, 236-0004 Japan; 4 Department of Emergency and Critical Care Medicine, Wakayama Medical University, 811-1 Kimiidera, Wakayama, 641-8509 Japan; 5 ICU Collaboration Network (ICON), Tokyo, Japan; 6 Division of Trauma and Surgical Critical Care, Osaka General Medical Center, 3-1-56 Bandaihigashi, Sumiyoshi-ku, Osaka, 558-8558 Japan; 7 Department of Emergency and Critical Care Medicine, Graduate School of Biomedical and Health Sciences, Hiroshima University, 1-2-3 Kasumi, Minami-ku, Hiroshima, 734-8551 Japan; 8 Department of Intensive Care Medicine, Kyoto Prefectural University of Medicine, 465 Kawaramachidori Hirokojiagarukajiicho, Kamigyo-ku, Kyoto, 602-8566 Japan; 9 Division of General Internal Medicine, Jichi Medical University Hospital, 3311-1 Yakushiji, Shimotsuke, Tochigi 329-0498 Japan; 10 Department of Health Services Research, Graduate School of Medicine, The University of Tokyo, 7-3-1 Hongo, Bunkyo-ku, Tokyo, 113-0033 Japan; 11 Department of Emergency and Critical Care Medicine, Kansai Medical University Medical Center, 10−15 Fumizonocho, Moriguchi, Osaka 570-8507 Japan; 12 Department of Critical Care Medicine, Yao Tokushukai General Hospital, 1-17 Wakakusacho, Yao, Osaka 581-0011 Japan; 13 Department of Anesthesia and Critical Care, Kobe City Medical Center General Hospital, 2-1-1 Minatojimaminamimachi, Chuo-ku, Kobe, 650-0047 Japan; 14 Acute Care Medical Center, Hyogo Prefectural Kakogawa Medical Center, 203 Kannochokanno, Kakogawa, Hyogo 675-0003 Japan; 15 Department of Emergency and Critical Care Medicine, Saitama Red Cross Hospital, 1-5 Shintoshin, Chuo-ku, Saitama, 330-8553 Japan; 16 Department of Anesthesiology and Intensive Care Medicine, Osaka University Graduate School of Medicine, 2-15 Yamadaoka, Suita, Osaka 565-0871 Japan; 17 Department of Emergency and Critical Care Medicine, Chiba University Graduate School of Medicine, 1-8-1 Inohana, Chuo-ku, Chiba, 260-8677 Japan; 18 Department of Anesthesiology and Intensive Care Medicine, University of the Ryukyus Hospital, 1076 Kiyuna, Ginowan, Okinawa 901-2725 Japan; 19 Department of Emergency and Critical Care Medicine, Japanese Red Cross Kyoto Daiichi Hospital, 15-749 Honmachi, Higashiyama-ku, Kyoto, 605-0981 Japan; 20 Department of Intensive Care Medicine, Asahi General Hospital, 1326 I, Asahi, Chiba 289-2511 Japan; 21 Japanese Red Cross Maebashi Hospital, Department of Critical Care and Emergency Medicine, 389-1 Asakuramachi, Maebashi, Gunma 371-0811 Japan; 22 Advanced Emergency and Critical Care Center, Yokohama City University Medical Center, 4-57 Urafunecho, Minami-ku, Yokohama, 232-0024 Japan; 23 Department of Emergency and Critical Care, National Hospital Organization Kumamoto Medical Center, 1-5 Ninomaru, Chuo-ku, Kumamoto, 860-0008 Japan; 24 Critical Care and Emergency Center, Metropolitan Tama General Medical Center, 2-8-29 Musashidai, Fuchu, Tokyo 183-8524 Japan; 25 Department of Anesthesiology and Critical Care Medicine, Fujita Health University School of Medicine, 1-98 Kutsukakecho, Toyoake, Aichi 470-1192 Japan; 26 Department of Intensive Care Medicine, Yokohama City Minato Red Cross Hospital, 3-12-1 Shinyamashita, Naka-ku, Yokohama, 231-8682 Japan; 27 Department of Anesthesiology, Fukushima Medical University, 1 Hikarigaoka, Fukushima, 960-1295 Japan; 28 Department of Emergency & Critical Care Medicine, National Hospital Organization Kyoto Medical Center, 1-1 Fukakusamukaihatacho, Fushimi-ku, Kyoto, 612-8555 Japan; 29 Department of Thoracic Surgery, Tokyo Metropolitan Institute for Geriatrics and Gerontology, 35-2 Sakaecho, Itabashi-ku, Tokyo, 173-0015 Japan; 30 Department of Emergency and Critical Care Medicine, Yodogawa Christian Hospital, 1-7-50 Kunijima, Higashiyodogawa-ku, Osaka, 533-0024 Japan; 31 Department of Emergency and Critical Care Center, Nagano Red Cross Hospital, 5-22-1 Wakasato, Nagano, 380-8582 Japan; 32 Intensive Care Unit, Shinshu University Hospital, 3-1-1 Asahi, Matsumoto, Nagano 390-8621 Japan; 33 Emergency and Disaster Medicine, Gifu University School of Medicine Graduate School of Medicine, 1-1 Yanagito, Gifu, 501-1112 Japan; 34 Department of Intensive Care Medicine, Tokyo Medical and Dental University, 1-5-45 Yushima, Bunkyo-ku, Tokyo, 113-0034 Japan; 35 Department of Anesthesiology, Tohoku University Hospital, 1-1 Seiryomachi, Aoba-ku, Sendai, 980-8574 Japan; 36 Intensive Care Unit, Kyushu University Hospital, 3-1-1 Maidashi, Higashi-ku, Fukuoka, 812-8582 Japan

**Keywords:** COVID-19, Post-intensive care syndrome, Trajectory, Function, Quality of life, ECMO

## Abstract

**Background:**

Post-intensive care syndrome (PICS) affects the quality of life (QOL) of survivors of critical illness. Although PICS persists for a long time, the longitudinal changes in each component and their interrelationships over time both remain unclear. This multicenter prospective study investigated the 2-year trajectory of PICS and its components as well as factors contributing to deterioration or recovery in mechanically ventilated patients with coronavirus disease 2019 (COVID-19), and also attempted to identify possible countermeasures.

**Methods:**

Patients who survived COVID-19 requiring mechanical ventilation completed questionnaires on the Barthel index, Short-Memory Questionnaire, Hospital Anxiety and Depression Scale, and EuroQol 5 dimensions 5-level every six months over a two-year period. Scores were weighted to account for dropouts, and the trajectory of each functional impairment was evaluated with alluvial diagrams. The prevalence of PICS and factors impairing or restoring function were examined using generalized estimating equations considering trajectories.

**Results:**

Among 334 patients, PICS prevalence rates in the four completed questionnaires were 72.1, 78.5, 77.6, and 82.0%, with cognitive impairment being the most common and lower QOL being noted when multiple impairments coexisted. Physical function and QOL indicated that many patients exhibited consistent trends of either recovery or deterioration. In contrast, cognitive function and mental health revealed considerable variability, with many patients showing fluctuating ratings in the later surveys. Delirium was associated with worse physical and mental health and poor QOL, while prolonged ventilation was associated with poor QOL. Living with family was associated with the recovery of all functions and QOL, while extracorporeal membrane oxygenation (ECMO) was associated with the recovery of cognitive function and mental health.

**Conclusions:**

Critically ill patients had PICS for a long period and followed different trajectories for each impairment component. Based on trajectories, known PICS risk factors such as prolonged ventilation and delirium were associated with impaired recovery, while ECMO and the presence of family were associated with recovery from PICS. In critically ill COVID-19 patients, delirium management and family interventions may play an important role in promoting recovery from PICS.

*Trial registration number:* UMIN000041276, August 01, 2020.

**Supplementary Information:**

The online version contains supplementary material available at 10.1186/s40560-025-00777-z.

## Background

Functional disabilities that occur during an intensive care unit (ICU) stay or after ICU or hospital discharge include physical, cognitive, and mental impairments, also known as post-intensive care syndrome (PICS), which affect the long-term prognosis and quality of life (QOL) of patients who survive to ICU discharge [1]. Despite the various reported risk factors for PICS, the prevalence remains high even with preventive measures, becoming an important social issue due [2–4].

Different recovery trajectories exist for functional impairments after ICU discharge [5]. Each component of PICS, including physical function, cognitive function, and mental health, follows a distinct trajectory, often characterized by complex impairments [6, 7]. A systematic review of responses after major stress events identified four main trajectories: resilience, recovery, chronic stress, and delayed onset [8]. Therefore, each component of PICS follows a different trajectory with complex impairments. However, a more detailed understanding of changes in these components will provide important information on when to assess and intensify interventions because surviving patients cannot be followed up many times after ICU discharge.

Minimizing the impact of PICS and promoting recovery requires a multifaceted approach. Comprehensive interventions were previously suggested to be effective at preventing and managing PICS [9]. However, the impact of specific respiratory therapies, such as prone positioning and extracorporeal membrane oxygenation (ECMO), and the use of continuous neuromuscular blocking agents on PICS in patients requiring mechanical ventilation remains unclear [10–12].

We herein conducted a multicenter, prospective study on patients with coronavirus disease 2019 (COVID-19) requiring mechanical ventilation, followed them every six months for two years, and tracked the evolution of each component of PICS. To the best of our knowledge, there have been no large-scale epidemiological studies that assessed long-term changes in patients requiring mechanical ventilation. Furthermore, modifiable factors related to PICS have yet to be examined, which may provide useful information for PICS measures in patients requiring mechanical ventilation.

## Methods

### Study design and setting

This study forms part of the multicenter observational study “Post-intensive care outcomes in patients with Coronavirus Disease 2019 study” (PICS-COVID study), which was conducted in collaboration with the Cross ICU Searchable Information System (CRISIS), the national registry in Japan for ICU patients with COVID-19 who require mechanical ventilation or ECMO, covering 80% of ICU beds throughout Japan [13].

All patients with COVID-19 admitted to 32 ICUs were considered for this study. A central office was established for the performance of administrative tasks, which included mailing questionnaires to patients, collecting and tabulating responses in the questionnaires, and handling inquiries from patients. Details on the participating institutions and the central office have been described in previous studies [14].

Approval for this study was granted by the Institutional Review Board of the National Hospital Organization Tokyo Medical Center (date: November 26, 2020, approval number: R20-133) and the Review Boards of each participating hospital. The study protocol was registered in the University Hospital Medical Information Network (UMIN000041276, date: August 01, 2020).

### Study population and eligibility criteria

The PICS survey was conducted among patients discharged from the ICU between March 2020 and December 2020. Inclusion criteria for the present study included patients with COVID-19 aged ≥ 20 years who required invasive mechanical ventilation during hospitalization. Indications for invasive mechanical ventilation management were selected at the discretion of the participating institutions. Severe acute respiratory syndrome coronavirus 2 (SARS-CoV-2) infection was confirmed using a polymerase chain reaction test. We excluded patients from whom written informed consent was not obtained and those who were unable to walk on their own before admission regardless of the use of assistive devices. All patients with COVID-19 who required mechanical ventilation were promptly registered in CRISIS in accordance with national policy when they were admitted to the ICU of each participating institution. Patients registered in CRISIS were enrolled in the present study if they met the inclusion criteria. Written informed consent was obtained from all patients.

### Procedures

Surveys to evaluate PICS were conducted four times, with questionnaires being sent to patients in February and October 2021 and April and October 2022. Participants were asked to respond to questions regarding the presence of dyspnea, weight loss, executive dysfunction, anxiety, and stress, and a subjective evaluation of their physical, cognitive, and mental status on a visual analog scale (VAS) ranging from 1 to 10 points. The Barthel index (BI) [15, 16], Short-Memory Questionnaire (SMQ) [17], Hospital Anxiety and Depression Scale (HADS)-anxiety, HADS-depression [18], and EuroQol 5 dimensions 5-level (EQ-5D-5L) [19] were used to assess physical function, cognitive function, mental health, and QOL, respectively. The results of the survey were fed back to the representatives of the participating facilities before the next questionnaire was mailed; however, no interventions were conducted based on the survey. Responses provided from a proxy approved by the patient to act in their place were permitted. Responses were collected and tabulated at the central office. Patients who responded to the survey were given an incentive worth 1,000 yen per survey.

### Variables and measurements

Patient characteristics were selected based on previous studies. Patient characteristics were as follows: age [20], sex [20], obesity defined as a body mass index (BMI) ≥ 25 [21], frailty defined as ≥ 4 on the clinical frailty scale [22], living with family [23], sequential organ failure assessment (SOFA) score [24], delirium [25], duration of mechanical ventilation [24], receipt of ECMO [26], tracheostomy [27], prone position [28], continuous neuromuscular blocking agent [24], maximum prednisolone equivalent daily dose [29], and rehabilitation program in the ICU conducted by a physical therapist [30].

### Outcomes

The primary outcome was risk factors for BI, SMQ, HADS, and EQ-5D-5L, taking into account the trajectory of the functional assessment over two years. Secondary outcomes were the prevalence of PICS after ICU discharge as indicated by the first, second, third, and fourth PICS surveys, and the prevalence of the three components of PICS. For the purposes of the present study, PICS was defined as any one of the following functional impairments: physical impairment defined as a BI score ≤ 90 [31], cognitive impairment as a SMQ score < 40 [32], or mental impairment as follows. Anxiety was defined as a score on the HADS-anxiety scale ≥ 8, while depression was defined as a score on the HADS-depression scale ≥ 8, when either anxiety or depression was met [33, 34]. To assess the details of the trajectory in physical function, BI items were classified into self-care, excretion, transferring and movement, as in the functional independence measure [35], and a functional impairment was defined if the score was not perfect in that classification.

### Statistical analysis

The demographic characteristics of patients were presented as medians and interquartile ranges, and results were shown as means and standard deviations for continuous variables. Since missing data in longitudinal data introduces a selection bias, we performed the stabilized inverse probability of censoring weights. The stabilized inverse probability of censoring weights at each follow-up questionnaire was generated using a logistic regression predicting the probability of non-missingness at the time point. Covariates were age, sex, BMI, the SOFA score, clinical frailty scale, comorbidities (hypertension, diabetes mellitus, cardiac disease, chronic kidney disease, autoimmune disease, malignant tumors, chronic obstructive pulmonary disease, and immunodeficiency), reintubation, ECMO, tracheostomy, continuous neuromuscular blocking agent, prone position, maximum prednisolone equivalent daily dose, continuous renal replacement therapy, intermittent renal replacement therapy, rehabilitation, delirium, the duration of mechanical ventilation, the length of ICU stay, the length of hospital stay, the presence of family members, and the ICU diary. To take the time course into account, we performed a multivariable logistic regression with generalized estimating equations adjusting for patient characteristics and time points. The patient characteristics were above variables in the Variables and Measurements section. We estimated the impact of a 10-year increase in age on outcomes. Similarly, we estimated the impact of a 7-day longer ventilator period and a 50 mg increase in steroid dose on outcomes. In addition, we performed sensitivity analysis using a multiple imputation method to confirm the robustness of main results. Twenty imputed datasets were created and estimates and standard errors were combined according to the Rubin's rule. The results were then denoted by the coefficient and 95% confidence interval. To visualize the trajectories of BI, SMQ, HADS, and EQ-5D-5L values, they are shown in an alluvial diagram by ventilator duration. Questionnaire response comparisons with and without ECMO were performed using the Student’s *t*-test. A p value < 0.05 (two-sided) was considered to be significant. Alluvial plots were analyzed using Python, version 3.12.1 (Python Software Foundation, Wilmington, Delaware, USA) software and all other data were examined using STATA SE software, version 17 (Stata Corp, College Station, TX, USA).

## Results

**Fig. 1:**
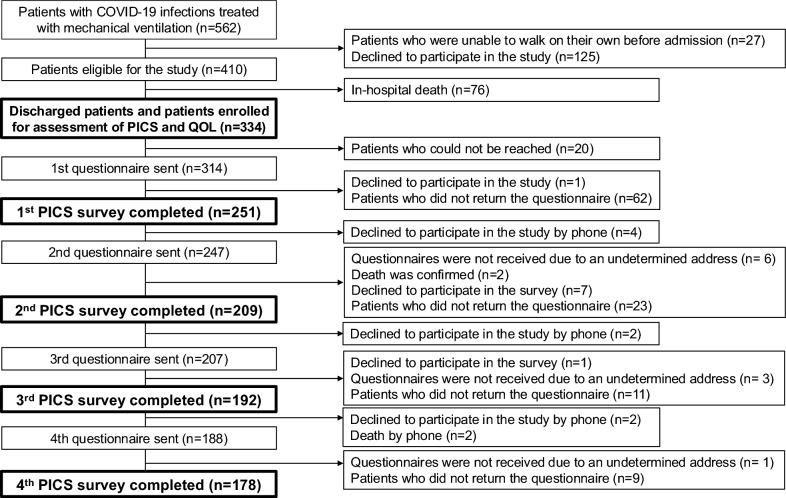
Study outline. Flow chart depicting the enrolment of subjects in the present study. *COVID-19* coronavirus disease 2019, *ICU* intensive care unit; *PICS* post-intensive care syndrome, *QOL* quality of life

The study outline is shown in Fig. 1. During the study period, we identified 562 patients treated with mechanical ventilation, 410 of whom had the ability to walk before admission and were enrolled in this study. Seventy-six patients died in hospital, while 334 were discharged alive and enrolled for the assessment of PICS and QOL. The first survey was completed by 251 patients, the second by 209, the third by 192, and 178 completed all four surveys. The mean (standard deviation) durations of survey responses after the date of ICU discharge were 5.5 (3.1), 12.5 (3.1), 18.5 (3.1), and 24.5 (3.1) months, respectively. The percentages of survey responses were 79.9% (251/314), 84.6% (209/247), 92.8% (192/207), and 94.7% (178/188), respectively.

The characteristics of patients enrolled for the assessment of PICS and QOL are shown in Table 1. Median age was 67 years (interquartile range: IQR, 58–74), the percentage of males was 79.6%, BMI was 25.4 kg/m^2^ (IQR, 22.6–28.7), and the percentage of patients who lived with family members was 74.9%. The percentage of patients who developed delirium during the ICU stay was 18.9%, and the duration of mechanical ventilation was 8 days (IQR, 6–14). Hypertension and diabetes mellitus were the most common comorbidities. Tracheostomy was performed on 18.0% of patients, and ECMO was introduced for 13.8%. Approximately 50% of patients were treated in the prone position, with a neuromuscular blocking agent, and rehabilitation. The percentage of patients treated with steroids was 77.5%. The characteristics of patients who responded to all four questionnaires are shown in Supplemental Table 1. The characteristics of patients who dropped out and the number of missing questionnaire values for each survey are presented in Supplemental Table 2 and 3.

**Table 1:** Patient characteristics and clinical course

	n = 334
Age, years, median (IQR)	67 (58, 74)
Male, n (%)	266 (79.6)
BMI, kg/m^2^, median (IQR)	25.4 (22.6, 28.7)
Obesity (BMI ≥ 25), n (%)	175 (52.4)
Living with family, n (%)	250 (74.9)
SOFA score on the day of ventilation start, median (IQR)	5 (4, 7)
PaO_2_/F_I_O_2_ ratio before mechanical ventilation	129 (91, 184)
Clinical frailty scale	2 (1, 3)
Frailty (Clinical frailty scale ≥ 4), n (%)	21 (6.3)
ICU mobility scale	
3 days	0 (0, 0)
5 days	0 (0, 1)
7 days	0 (0, 1)
Delirium, n (%)	63 (18.9)
Duration of delirium within 1 week of ICU admission, day, median (IQR)	2 (1, 4)
Duration of mechanical ventilation, day, median (IQR)	8 (6, 14)
Length of ICU stay, day, median (IQR)	11 (8, 18)
Length of hospital stay, day, median (IQR)	21 (11, 38)
Comorbidity, n (%)	225 (67.4)
Hypertension	146 (43.7)
Diabetes mellitus	114 (34.1)
Cardiac disease	37 (11.1)
COPD	30 (9.0)
CKD G5	9 (2.7)
Autoimmune disorder	12 (3.6)
Immunodeficiency	12 (3.6)
Malignant tumor	22 (6.6)
Reintubation, n (%)	12 (3.6)
Tracheostomy, n (%)	60 (18.0)
ECMO, n (%)	46 (13.8)
Duration of ECMO, day, median (IQR)	11.5 (9, 17)
Prone position, n (%)	154 (46.1)
Time from ICU admission to prone position, day, median (IQR)	1 (1, 2)
Duration of prone position, day, median (IQR)	4 (2, 5)
Prone position time per session, hour, median (IQR)	16 (8, 16)
Continuous neuromuscular blocking agent, n (%)	154 (46.1)
Duration of continuous neuromuscular blocking agent, day, median (IQR)	2 (2, 4)
Corticosteroid, n (%)	259 (77.5)
Maximum prednisolone dose, mg/day, median (IQR)	44 (30, 82.5)
RRT, n (%)	29 (8.7)
IRRT	16 (4.8)
CRRT	24 (7.2)
Rehabilitation program, n (%)	182 (54.5)
Time from ICU admission to rehabilitation program initiation, day, median (IQR)	5 (2, 15)
ICU diary, n (%)	36 (10.8)

*BMI* body mass index, *CKD* chronic kidney disease, *COPD* chronic obstructive pulmonary disease, *CRRT* continuous renal replacement therapy, *ECMO* extracorporeal membrane oxygenation, *ICU* intensive care unit, *IQR* interquartile range, *IRRT* intermittent RRT, *RRT* renal replacement therapy, *SOFA* sequential organ failure assessment

**Table 2:** Questionnaire results

	1st survey (n = 251)	2nd survey (n = 209)	3rd survey (n = 192)	4th survey (n = 178)
**Physical function**				
Barthel index, mean (SD)	92.4 (33.8)	94.7 (44.8)	93.6 (37)	94.4 (37.1)
Self-care impairment, n (%)	41 (16.3)	25 (12.0)	16 (8.3)	16 (9.0)
Excretion impairment, n (%)	37 (14.7)	23 (11.0)	26 (13.5)	23 (12.9)
Transferring impairment, n (%)	20 (8.0)	9 (4.3)	10 (5.2)	8 (4.5)
Movement impairment, n (%)	39 (15.5)	33 (15.8)	22 (11.5)	22 (12.4)
**Cognitive function**				
Short-Memory Questionnaire, mean (SD)	38.2 (13.9)	38.0 (20.2)	36.4 (15.4)	36.9 (15.7)
**Mental health**				
Anxiety (HADS-Anxiety score ≥ 8), n (%)	49 (19.5)	25 (12.0)	34 (17.7)	25 (14.0)
Depression (HADS-Depression score ≥ 8), n (%)	49 (19.5)	35 (16.8)	38 (19.8)	36 (20.2)
HADS score, mean (SD)	9.5 (8.4)	9.1 (12.0)	9.6 (10.0)	8.8 (8.0)
HADS-Anxiety score	4.6 (4.3)	4.3 (5.9)	4.6 (5.2)	3.8 (3.9)
HADS-Depression score	4.8 (4.7)	4.8 (6.5)	5.1 (5.4)	4.9 (4.8)
**QOL**				
EQ-5D-5L, mean (SD)	0.804 (0.336)	0.839 (0.410)	0.833 (0.356)	0.824 (0.359)
**Visual analog scale, mean (SD)**				
Physical function (on a scale of 1 to 10)	6.9 (2.2)	7.1 (2.0)	7.1 (2.0)	7.1 (1.9)
Cognitive function (on a scale of 1 to 10)	8.2 (2.1)	8.0 (1.9)	7.9 (1.9)	8.0 (1.8)
Mental health (on a scale of 1 to 10)	7.6 (2.3)	7.6 (2.3)	7.7 (2.2)	7.8 (1.9)
**Others, n (%)**				
Dyspnea	118 (47.0)	96 (45.9)	89 (46.4)	89 (50.0)
Walking difficulty	89 (35.5)	54 (25.8)	55 (28.7)	57 (32.0)
Weight loss	154 (61.4)	48 (23.0)	39 (20.3)	48 (27.0)
Memory impairment	74 (29.5)	66 (31.6)	66 (34.4)	68 (38.2)
Executive dysfunction	120 (47.8)	93 (44.5)	96 (50.0)	82 (46.1)
Depression	103 (41.0)	81 (38.8)	71 (37.0)	70 (39.3)
Anxiety	144 (57.4)	107 (51.2)	94 (49.0)	93 (52.3)
Sleeping disorder	113 (45.0)	92 (44.0)	82 (42.7)	73 (41.0)

*EQ-5D-5L* EuroQol 5 dimensions 5-level, *HADS* Hospital Anxiety and Depression Scale, *QOL* quality of life, *SD* standard deviation

Outcome scores of Barthel index, Short-Memory Questionnaire, Hospital Anxiety and Depression Scale-Anxiety score, Hospital Anxiety and Depression Scale-Depression score, and EuroQol 5 dimensions 5-level were stabilized using inverse probability of censoring weights that take into account missing data. The included covariates were age, gender, BMI, SOFA score, clinical frailty scale, comorbidities (hypertension, diabetes, cardiac disease, chronic renal disease, autoimmune disease, malignancy, chronic obstructive pulmonary disease, immunodeficiency), reintubation, ECMO, tracheostomy, continuous neuromuscular blockade, supine position, maximum prednisolone equivalent daily dose, continuous renal replacement therapy, intermittent renal replacement therapy, rehabilitation, delirium, duration of mechanical ventilation, length of ICU stay, length of hospital stay, ICU duration, presence of family members, and use of an ICU diary

**Fig. 2:**
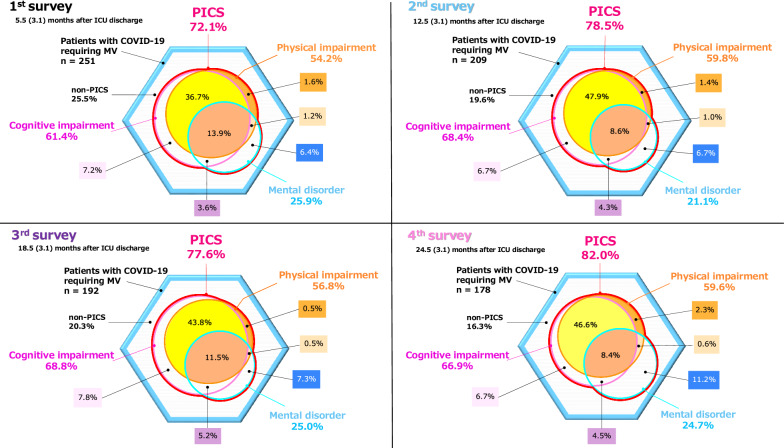
The prevalence of post-intensive care syndrome after intensive care unit discharge in first, second, third, and fourth surveys**.** Distribution of the prevalence of post-intensive care syndrome (PICS) across each survey among patients with coronavirus disease 2019 (COVID-19) who required ventilatory management during admission. Outcome scores were stabilized using inverse probability of censoring weights that take into account missing data. The included covariates were age, sex, BMI, the SOFA score, clinical frailty scale, comorbidities (hypertension, diabetes mellitus, cardiac disease, chronic kidney disease, autoimmune disease, malignant tumors, chronic obstructive pulmonary disease, and immunodeficiency), reintubation, ECMO, tracheostomy, continuous neuromuscular blocking agent, prone position, maximum prednisolone equivalent daily dose, continuous renal replacement therapy, intermittent renal replacement therapy, rehabilitation, delirium, the duration of mechanical ventilation, the length of ICU stay, the length of hospital stay, the presence of family members, and use of an ICU diary. The first survey included 251 patients with a mean (standard deviation [SD]) time since ICU discharge of 5.5 (3.1) months, the second survey included 209 patients with a mean (SD) time since ICU discharge of 12.5 (3.1) months, the third survey included 192 patients with a mean (SD) time since ICU discharge of 18.5 (3.1) months, and the fourth survey included 178 patients with a mean (SD) time since ICU discharge of 24.5 (3.1) months. COVID-19, coronavirus disease 2019; ICU, intensive care unit; PICS, post-intensive care syndrome

**Fig. 3:**
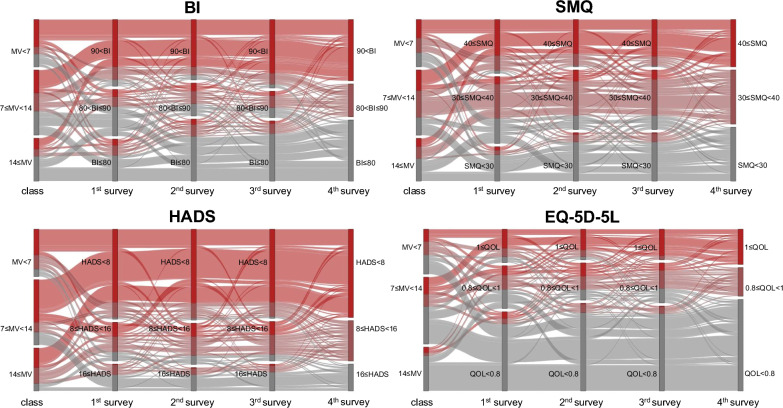
An alluvial diagram of physical impairment, cognitive impairment, mental health, and quality of life. The Barthel index (BI), Short-Memory Questionnaire (SMQ), Hospital Anxiety and Depression Scale (HADS)-anxiety, HADS-depression, and EuroQol 5 dimensions 5-level (EQ-5D-5L) were used to assess physical function, cognitive function, mental health, and QOL, respectively. The alluvial diagram illustrates the transitions in scores for each assessment, classified into three groups based on the duration of mechanical ventilation: less than 7 days, 7 to 14 days, and more than 14 days, among patients who completed all four surveys (n = 178). Outcome scores were stabilized using inverse probability weighting to account for missing data. The included covariates were age, sex, BMI, SOFA score, clinical frailty scale, comorbidities (hypertension, diabetes, cardiac disease, chronic kidney disease, autoimmune disease, malignancy, chronic obstructive pulmonary disease, and immunodeficiency), reintubation, ECMO, tracheostomy, prolonged neuromuscular blockade, prone positioning, maximum daily equivalent dose of prednisolone, continuous renal replacement therapy, intermittent renal replacement therapy, rehabilitation, delirium, duration of mechanical ventilation, ICU stay, hospital stay, presence of family, and use of an ICU diaries. For each assessment, patients classified into the best category at the fourth survey are represented by red bands, while those classified into the worst category are depicted by gray bands. The Barthel Index and EuroQol 5 Dimensions 5-Level (EQ-5D-5L) showed a prominent upward trend in red bands and a downward trend in black bands. These trends diminished in frequency and thickness in the later stages of the survey, indicating that many patients exhibited consistent trends of recovery or deterioration. In contrast, the Short-Memory Questionnaire and the Hospital Anxiety and Depression Scale revealed less discernible directional changes, with wider and more frequent fluctuations in the bands as the surveys progressed. This suggests that many patients experienced variability in their assessments. Furthermore, among patients requiring mechanical ventilation for more than 14 days, a higher proportion were classified in the gray (worst) category across all assessments by the fourth survey. However, as indicated by the limited presence of fluctuating bands, fewer patients exhibited variability in their scores

The prevalence of PICS among patients with severe COVID-19 in the first, second, third, and fourth surveys is shown in Fig. 2, and the mean EQ-5D-5L values by functional impairment are shown in Supplemental Fig. 1. Outcome scores of Barthel index, Short-Memory Questionnaire, Hospital Anxiety and Depression Scale-Anxiety score, Hospital Anxiety and Depression Scale-Depression score, and EuroQol 5 dimensions 5-level were stabilized using inverse probability of censored weighting to account for missing data. The following covariates were included: age, gender, BMI, SOFA score, clinical frailty scale, comorbidities (hypertension, diabetes, cardiac disease, chronic renal disease, autoimmune disease, malignancy, chronic obstructive pulmonary disease, immunodeficiency), reintubation, ECMO, tracheostomy, continuous neuromuscular blockade, supine position, maximum prednisolone equivalent daily dose, continuous renal replacement therapy, intermittent renal replacement therapy, rehabilitation, delirium, duration of mechanical ventilation, length of ICU stay, length of hospital stay, ICU duration, presence of family members, and use of an ICU diary. PICS prevalence rates in the four surveys gradually increased: 72.1, 78.5, 77.6, and 82.0%, respectively. In all questionnaires, the most common functional impairment was cognitive impairment, followed by physical impairment and mental impairment. The prevalence of each component did not show a consistent change over time. Two or more functional disabilities were present at the same time in 55.4, 61.7, 60.9, and 60.1%, respectively. EQ-5D-5L values, indicative of QOL, were slightly lower when multiple functional impairments were present. The prevalence of PICS in patients who responded to all four questionnaires is shown in Supplemental Fig. 2, and the mean EQ-5D-5L values by functional impairment are shown in Supplemental Fig. 3. An alluvial diagram showing changes in physical function, cognitive function, mental health, and QOL in patients classified as having a ventilation duration of less than 7 days, between 7 and 14 days, and more than 14 days is shown in Fig. 3. This figure illustrates the transitions in BI, SMQ, HADS, and EQ-5D-5L scores across each survey among complete cases, excluding patients with missing responses. Outcome scores were stabilized using inverse probability of censored weighting to account for missing data. The included covariates were age, sex, BMI, SOFA score, clinical frailty scale, comorbidities (hypertension, diabetes, cardiac disease, chronic kidney disease, autoimmune disease, malignancy, chronic obstructive pulmonary disease, and immunodeficiency), reintubation, ECMO, tracheostomy, prolonged neuromuscular blockade, prone positioning, maximum daily equivalent dose of prednisolone, continuous renal replacement therapy, intermittent renal replacement therapy, rehabilitation, delirium, duration of mechanical ventilation, ICU stay, hospital stay, presence of family, and use of an ICU diaries. Focusing on the red bands, which indicate favorable outcomes, and the gray bands, which represent unfavorable outcomes in the final assessment for physical function and QOL, it is evident that the frequency of upward and downward changes diminishes toward the later surveys. This suggests that many patients exhibited consistent trends of either recovery or deterioration. In contrast, for cognitive function and mental health, the bands exhibiting variability remain prominent even in the later stages, indicating that a considerable proportion of patients experienced fluctuating evaluations until the later surveys. Furthermore, among patients requiring mechanical ventilation for more than 14 days, a higher proportion demonstrated unfavorable outcomes across all assessments in the final evaluation. However, fluctuating evaluations were less frequent in this group.

Table 2 shows responses to the questionnaires in each survey. Anxiety, dyspnea, and executive dysfunction were the most common subjective symptoms. Weight loss was the most common complaint in the first survey, but markedly decreased over time. Physical-related symptoms generally improved over time, whereas cognitive and psychiatric-related symptoms did not. Furthermore, there were no obvious changes in VAS. Detailed results on BI showed that items related to self-care generally improved, while movement and transferring showed delayed recovery and excretion-related disorders were less likely to improve. Supplemental Table 4 shows responses to the questionnaires in each survey by patients who responded to all four questionnaires.

**Fig. 4:**
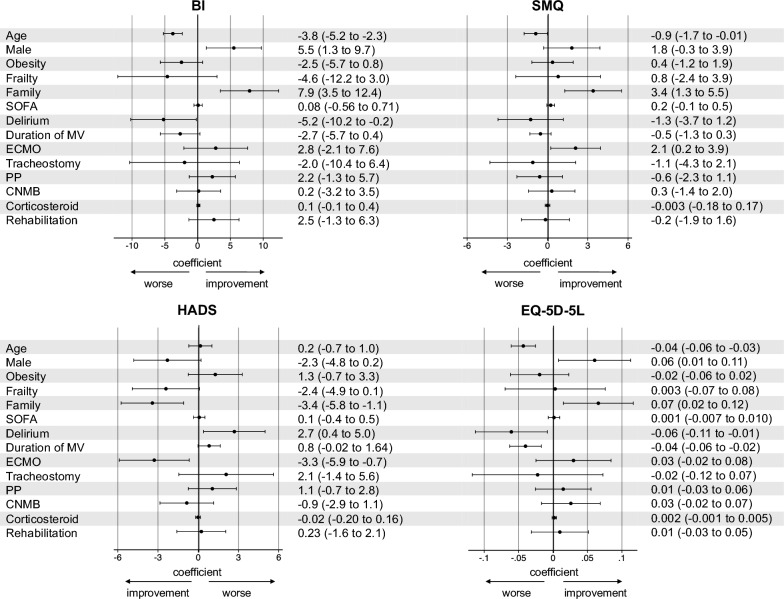
Analysis of generalized estimating equations The results of a generalized estimating equations analysis are shown in a forest plot. The results were then denoted by the coefficient and 95% confidence interval. Age was calculated in increments of 10 years, the duration of mechanical ventilation in increments of 7 days, and corticosteroid doses in increments of 50 mg/day. *BI* Barthel index, *CNMB* continuous neuromuscular blocking agent, *ECMO* extracorporeal membrane oxygenation, *EQ-5D-5L* EuroQol 5 dimensions 5-level, *HADS* Hospital Anxiety and Depression Scale, *MV* mechanical ventilation; *PICS* post-intensive care syndrome, *PP* prone position, *SMQ* Short-Memory Questionnaire, *SOFA* sequential organ failure assessment

The results of a generalized estimating equations analysis are shown in a forest plot (Fig. 4). Age (per 10-year increase) correlated with worse BI (-3.8; -5.2 to -2.3), SMQ (-0.9; -1.7 to -0.01), and EQ-5D-5L (-0.04; -0.06 to -0.03). Males showed significant improvements in BI (5.5; 1.3 to 9.7) and EQ-5D-5L (0.06; 0.01 to 0.11). Living with family showed significant improvements in BI (7.9; 3.5 to 12.4), SMQ (3.4; 1.3 to 5.5), HADS (-3.4; -5.8 to -1.1), and EQ-5D-5L (0.07; 0.02 to 0.12). Delirium correlated with worse BI (-5.2; -10.2 to -0.2), HADS (2.7; 0.4 to 5.0), and EQ-5D-5L (-0.06; -0.11 to -0.01). Mechanical ventilation (per 7 days) correlated with worse EQ-5D-5L (-0.04; -0.06 to -0.02). ECMO showed significant improvements in SMQ (2.1; 0.2 to 3.9) and HADS (-3.3; -5.9 to -0.7). Patient backgrounds with and without ECMO are shown in Supplemental Table 5: patients in the ECMO group were significantly younger, had better activities of daily living (ADL) prior to admission, and were more severely ill. The results of sensitivity analysis using a multiple imputation method are shown in Supplemental Fig. 4. The results in the multiple imputation were similar to those in the main analysis.

## Discussion

In a 2-year PICS study on patients requiring mechanical ventilation, more than 70% of patients had PICS that persisted for a long time. The trajectory of PICS varied, with evaluations of cognitive function and mental health tending to fluctuate even in the later surveys. Regarding physical function, ADL decline requiring care generally improved, whereas excretion-related disabilities did not. Considering trajectories, age and delirium were factors that exacerbated functional impairment, while male sex, living with family, and receiving ECMO were associated with functional and recovery.

The strength of the present study on critical care is that it investigated changes in PICS over a two-year period for the first time based on simultaneous evaluations of physical function, cognitive function, mental health, and QOL. The results obtained revealed that many patients had PICS for a long time, which reduced QOL, and also that each PICS component had a different trajectory over two years. The physical and cognitive functions and QOL of critically ill patients change over time [5–8]. Therefore, future PICS research needs to examine the prevalence of PICS and risk factors at multiple time points and also take PICS measures based on trajectories. Previous studies suggested the effectiveness of multiple follow-up systems in follow-up clinics and telemedicine that intervene with PICS measures on multiple occasions as a post-discharge PICS intervention [36, 37]; however, there are also many different challenges [38–40]. Since different treatments and care at different time points are required for PICS, we need to be able to respond to patients flexibly with PICS follow-up systems.

One of the unique features of the results of the present study is that most critically ill patients on ECMO achieved functional recovery when trajectories were considered. Patients on ECMO were generally younger and had better ADL before hospitalization, which clearly introduces confounding factors. Therefore, we believe that ECMO does not directly promote recovery from PICS. However, while there are reports indicating that ECMO is a significant risk factor for the development of PICS [24, 26], our results suggested that the patients on ECMO did not necessarily follow the worse PICS courses. Actually, some studies on the long-term outcomes of patients on ECMO have also reported improved functional assessments in those who have survived for extended periods [41, 42]. Such accumulating evidence may strengthen the confidence of patients, relatives, and ICU teams involved in the treatment of severe respiratory failure requiring ECMO support.

The incidence of delirium was lower in the present study than in previous studies on COVID-19 [43, 44], which may be attributed to differences in invasive mechanical ventilation rates and severe respiratory failure treatment approaches under the phases of a pandemic. The assessment of delirium was challenging in COVID-19 patients due to deep sedation, the prone position, and the use of neuromuscular blocking agents as a lung protection strategy [45]. Consistent with previous findings [25, 46–49], delirium was associated with worse physical and mental outcomes and lower QOL over time in our cohort, reaffirming the critical importance of delirium prevention and management strategies. Similarly, prolonged mechanical ventilation has been associated with decreased QOL [50], highlighting the potential importance of validated care bundles, such as the ABCDEF bundle, which have been shown to reduce the duration of mechanical ventilation [51]. Another important result of this survey, which considered trajectories, is that patients who lived with family members prior to hospitalization had better functional outcomes and QOL after discharge. Family visitation was associated with a reduced risk of developing delirium [52], and it is important from the perspective of preventing long-term functional impairment during the ICU stay. In addition, critically ill patients often require intensive rehabilitation and home care services [53]; therefore, family support may be critical to these efforts beyond their emotional support. Family involvement and cooperation in treatment, when available, were indispensable to the management of critically ill patients and patient-centered medical care for long-term recovery [54].

The prevalence of PICS slightly increased at 24 months, with a higher rate of cognitive impairment during the 2-year follow-up after ICU discharge. These results may have been affected by the characteristics of COVID-19 infection in the study cohort. SARS-CoV-2 is known to directly invade the nasal mucosa and lung tissue, causing a systemic inflammatory response and microvascular damage, leading to cerebral neuropathy and affecting cognitive function [55, 56]. Furthermore, corticosteroid administration as an acute treatment for COVID-19 may have adverse effects on the central nervous system, including cognitive impairment, sleep disturbance, and delirium [57]. Various long-term sequelae, including neurological disorders, difficulty concentrating, and fatigue, are known as long-COVID or post-acute COVID-19 [58, 59].

The generalizability of the present study needs to be carefully considered from the following two perspectives. There are limitations due to the specificity of COVID-19 described above. However, ICU patients are an originally heterogenous population in whom underlying diseases and severities vary widely. In this view, as this study focused only to ventilated patients with respiratory failure, the obtained results might be interpretated as the PICS with mechanically ventilated ICU patients. The majority of patients are Japanese, therefore, the results of the present study might have limited generalizability. There are several other limitations that need to be addressed. Difficulties were associated with assessing cognitive function and mental health in the acute phase because patients requiring ventilatory management were included. Since patients with the ability to walk unassisted were selected, their physical and cognitive functions had stabilized to some extent before hospitalization. However, some patients may have had organic mental disorder characteristics or mild cognitive dysfunction prior to the onset of COVID-19. Furthermore, since the present study only involved the assessment of outcomes obtained using self-reported measures, it was not possible to assess them in person; however, the self-reported measures used were validated [60]. Moreover, this study was based on mailed questionnaires, and questionnaire responses were allowed to be substituted by family members if the patient was unable to respond directly, which may have resulted in better or worse patient evaluations by family members. In addition, the percentage of family members who substituted was not surveyed. Another limitation is that the severity and sequelae of SARS-CoV-2 vary according to the variant form of the virus; however, this study did not investigate the variant form. Furthermore, PICS studies conducted using different assessment tools may not be comparable, and minor physical impairment may have been missed using BI. Additionally, although the weighting of questionnaire results based on patient backgrounds was a strength of the present study, patient selection bias was introduced due to drop-outs who did not complete the questionnaire. Further research in which these limitations are addressed is needed.

## Conclusions

A 2-year PICS study on patients requiring mechanical ventilation revealed that many patients had PICS that persisted long after their discharge from the hospital. The trajectory of PICS varied by function, and when trajectories were considered, age, a prolonged mechanical ventilation period, and delirium were identified as independent factors affecting functional impairment and QOL decline, while functional recovery was better in patients with severe COVID-19 on ECMO and those living with family. In critically ill patients with COVID-19, addressing delirium and implementing family-centered interventions may play a meaningful role in facilitating recovery from PICS.

## Supplementary Information

Supplementary Material 1.

Supplementary Material 2.

Supplementary Material 3.

Supplementary Material 4.

Supplementary Material 5.

## Data Availability

Individual participant data that underlie the results reported in this article are available from the corresponding author upon reasonable request.

## Abbreviations

ICU: Intensive care unit
PICS: Post-intensive care syndrome
QOL: Quality of life
ICU-AW: ICU-acquired weakness
ECMO: Extracorporeal membrane oxygenation
COVID-19: Coronavirus disease 2019
PICS-COVID study: Post-intensive care outcomes in patients with Coronavirus Disease 2019 study
CRISIS: Cross ICU Searchable Information System
SARS-CoV-2: Severe acute respiratory syndrome coronavirus 2
VAS: Visual analog scale
BI: Barthel index
SMQ: Short-Memory Questionnaire
HADS: Hospital anxiety and depression scale
EQ-5D-5L: EuroQol 5 dimensions 5-level
BMI: Body mass index
SOFA: Sequential organ failure assessment
IQR: Interquartile range
