# Supplementary material for: Two-year trajectory of functional recovery and quality of life in post-intensive care syndrome: a multicenter prospective observational study on mechanically ventilated patients with coronavirus disease-19
Source: J Intensive Care. 2025 Feb 6;13:7. doi: 10.1186/s40560-025-00777-z (PMC11800417; doi:10.1186/s40560-025-00777-z)
Supplement: Supplementary file 1 — Supplementary Material 1. [file 40560_2025_777_MOESM1_ESM.pdf]

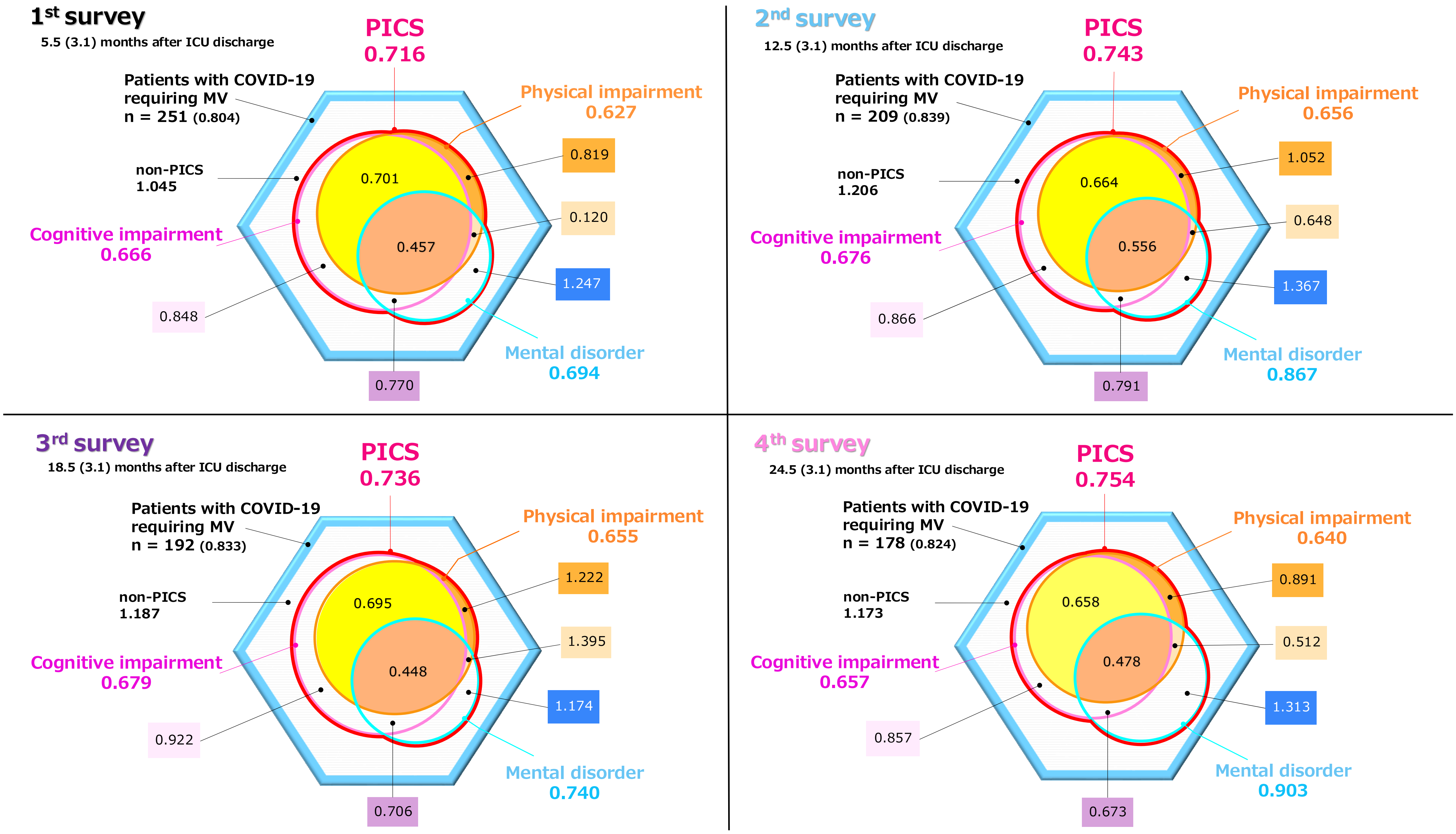
1st survey
5.5 (3.1) months after ICU discharge
PICS
0.716
Patients with COVID-19 requiring MV n = 251 (0.804)
Physical impairment 0.627
non-PICS 1.045
Cognitive impairment 0.666
Mental disorder 0.694
0.701
0.457
0.819
0.120
1.247
0.848
0.770
2nd survey
12.5 (3.1) months after ICU discharge
PICS
0.743
Patients with COVID-19 requiring MV n = 209 (0.839)
Physical impairment 0.656
non-PICS 1.206
Cognitive impairment 0.676
Mental disorder 0.867
0.664
0.556
1.052
0.648
1.367
0.866
0.791
3rd survey
18.5 (3.1) months after ICU discharge
PICS
0.736
Patients with COVID-19 requiring MV n = 192 (0.833)
Physical impairment 0.655
non-PICS 1.187
Cognitive impairment 0.679
Mental disorder 0.740
0.695
0.448
1.222
1.395
1.174
0.922
0.706
4th survey
24.5 (3.1) months after ICU discharge
PICS
0.754
Patients with COVID-19 requiring MV n = 178 (0.824)
Physical impairment 0.640
non-PICS 1.173
Cognitive impairment 0.657
Mental disorder 0.903
0.658
0.478
0.891
0.512
1.313
0.857
0.673
