# Supplementary material for: Two-year trajectory of functional recovery and quality of life in post-intensive care syndrome: a multicenter prospective observational study on mechanically ventilated patients with coronavirus disease-19
Source: J Intensive Care. 2025 Feb 6;13:7. doi: 10.1186/s40560-025-00777-z (PMC11800417; doi:10.1186/s40560-025-00777-z)
Supplement: Supplementary file 2 — Supplementary Material 2. [file 40560_2025_777_MOESM2_ESM.pdf]

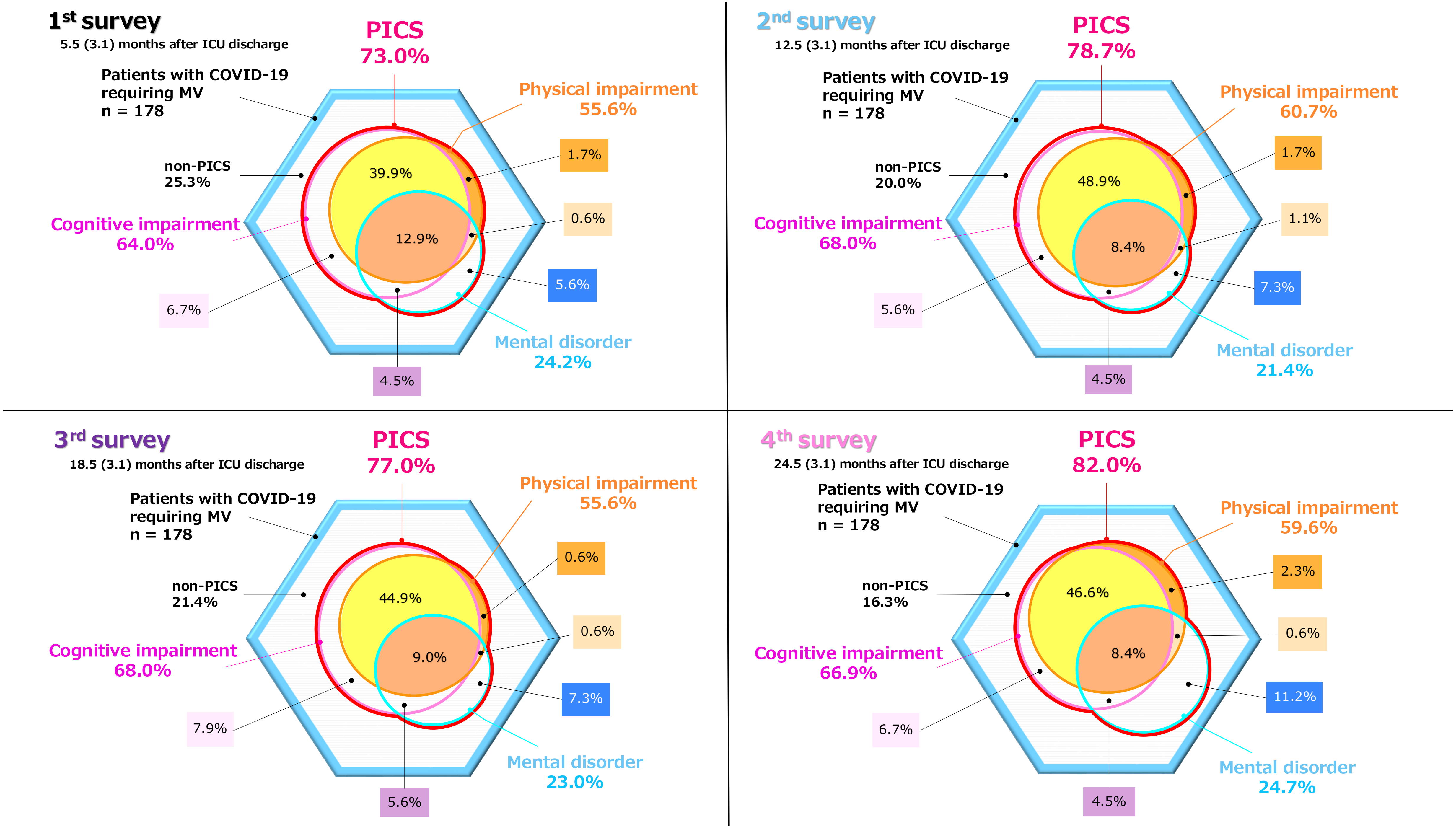
1st survey
5.5 (3.1) months after ICU discharge
PICS
73.0%
Patients with COVID-19
requiring MV
n = 178
Physical impairment
55.6%
non-PICS
25.3%
39.9%
1.7%
0.6%
Cognitive impairment
64.0%
12.9%
5.6%
6.7%
Mental disorder
24.2%
4.5%
2nd survey
12.5 (3.1) months after ICU discharge
PICS
78.7%
Patients with COVID-19
requiring MV
n = 178
Physical impairment
60.7%
1.7%
non-PICS
20.0%
48.9%
1.1%
Cognitive impairment
68.0%
8.4%
7.3%
5.6%
Mental disorder
21.4%
4.5%
3rd survey
18.5 (3.1) months after ICU discharge
PICS
77.0%
Patients with COVID-19
requiring MV
n = 178
Physical impairment
55.6%
0.6%
non-PICS
21.4%
44.9%
0.6%
Cognitive impairment
68.0%
9.0%
7.3%
7.9%
Mental disorder
23.0%
5.6%
4th survey
24.5 (3.1) months after ICU discharge
PICS
82.0%
Patients with COVID-19
requiring MV
n = 178
Physical impairment
59.6%
2.3%
non-PICS
16.3%
46.6%
0.6%
Cognitive impairment
66.9%
8.4%
11.2%
6.7%
Mental disorder
24.7%
4.5%
