# Supplementary material for: Two-year trajectory of functional recovery and quality of life in post-intensive care syndrome: a multicenter prospective observational study on mechanically ventilated patients with coronavirus disease-19
Source: J Intensive Care. 2025 Feb 6;13:7. doi: 10.1186/s40560-025-00777-z (PMC11800417; doi:10.1186/s40560-025-00777-z)
Supplement: Supplementary file 3 — Supplementary Material 3. [file 40560_2025_777_MOESM3_ESM.pdf]

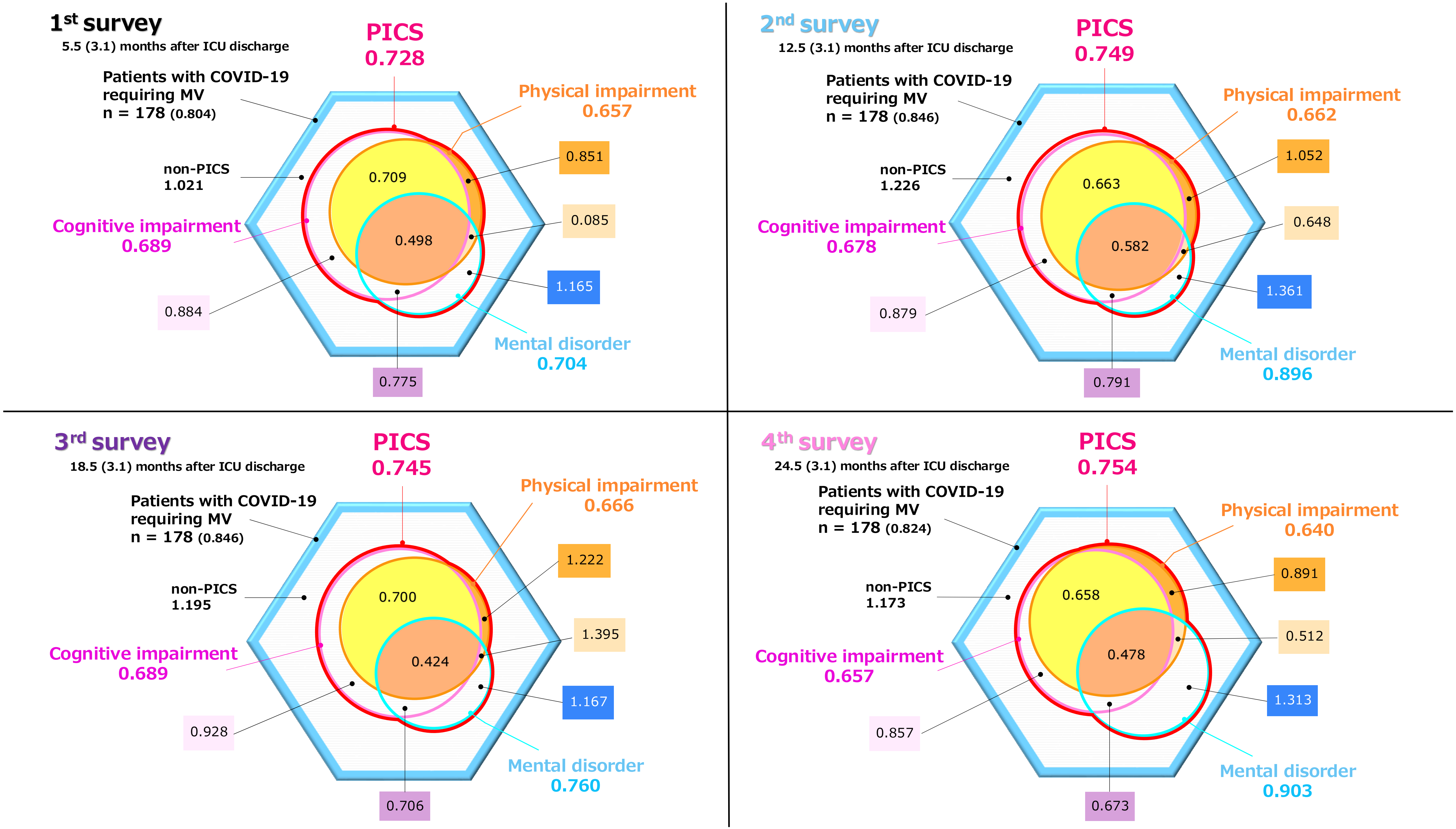
1st survey
5.5 (3.1) months after ICU discharge
PICS 0.728
Patients with COVID-19 requiring MV n = 178 (0.804)
Physical impairment 0.657
non-PICS 1.021
0.709
0.851
0.085
Cognitive impairment 0.689
0.498
1.165
0.884
Mental disorder 0.704
0.775
2nd survey
12.5 (3.1) months after ICU discharge
PICS 0.749
Patients with COVID-19 requiring MV n = 178 (0.846)
Physical impairment 0.662
non-PICS 1.226
0.663
1.052
0.648
Cognitive impairment 0.678
0.582
1.361
0.879
Mental disorder 0.896
0.791
3rd survey
18.5 (3.1) months after ICU discharge
PICS 0.745
Patients with COVID-19 requiring MV n = 178 (0.846)
Physical impairment 0.666
non-PICS 1.195
0.700
1.222
1.395
Cognitive impairment 0.689
0.424
1.167
0.928
Mental disorder 0.760
0.706
4th survey
24.5 (3.1) months after ICU discharge
PICS 0.754
Patients with COVID-19 requiring MV n = 178 (0.824)
Physical impairment 0.640
non-PICS 1.173
0.658
0.891
0.512
Cognitive impairment 0.657
0.478
1.313
0.857
Mental disorder 0.903
0.673
