# Supplementary figures and images for: Two-year trajectory of functional recovery and quality of life in post-intensive care syndrome: a multicenter prospective observational study on mechanically ventilated patients with coronavirus disease-19
Source: J Intensive Care. 2025 Feb 6;13:7. doi: 10.1186/s40560-025-00777-z (PMC11800417; doi:10.1186/s40560-025-00777-z)

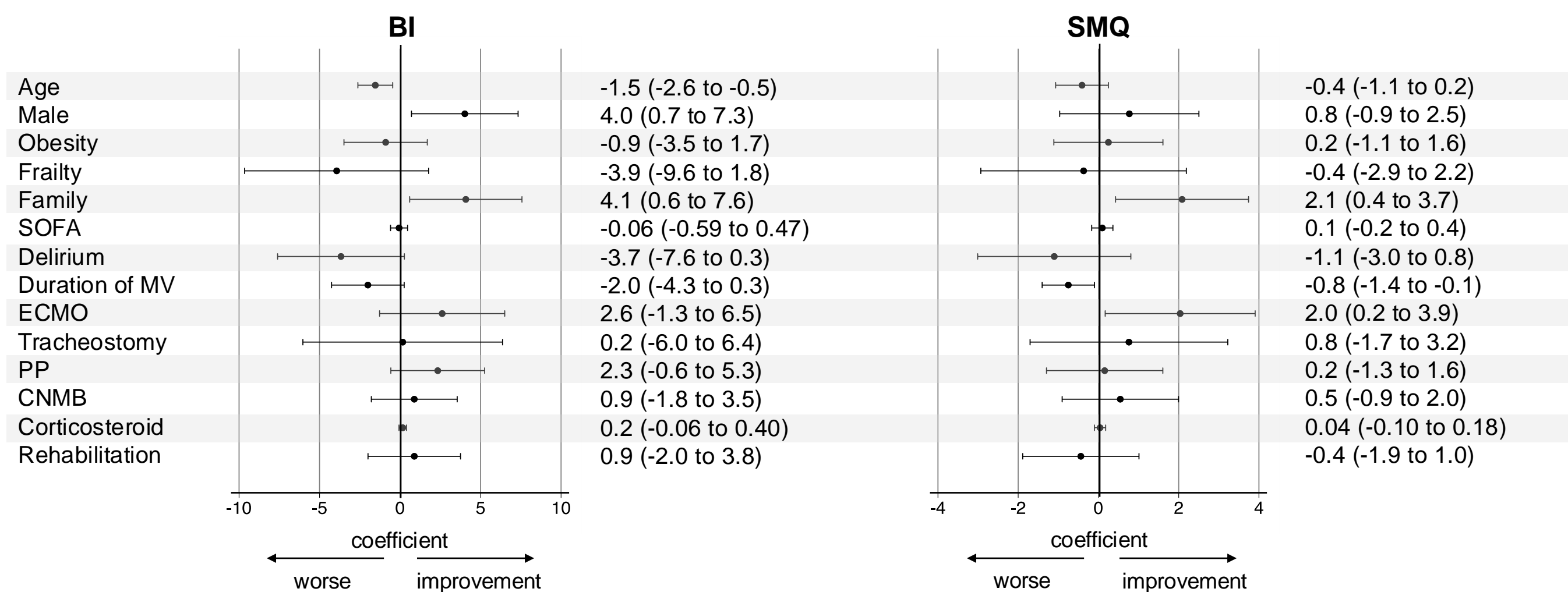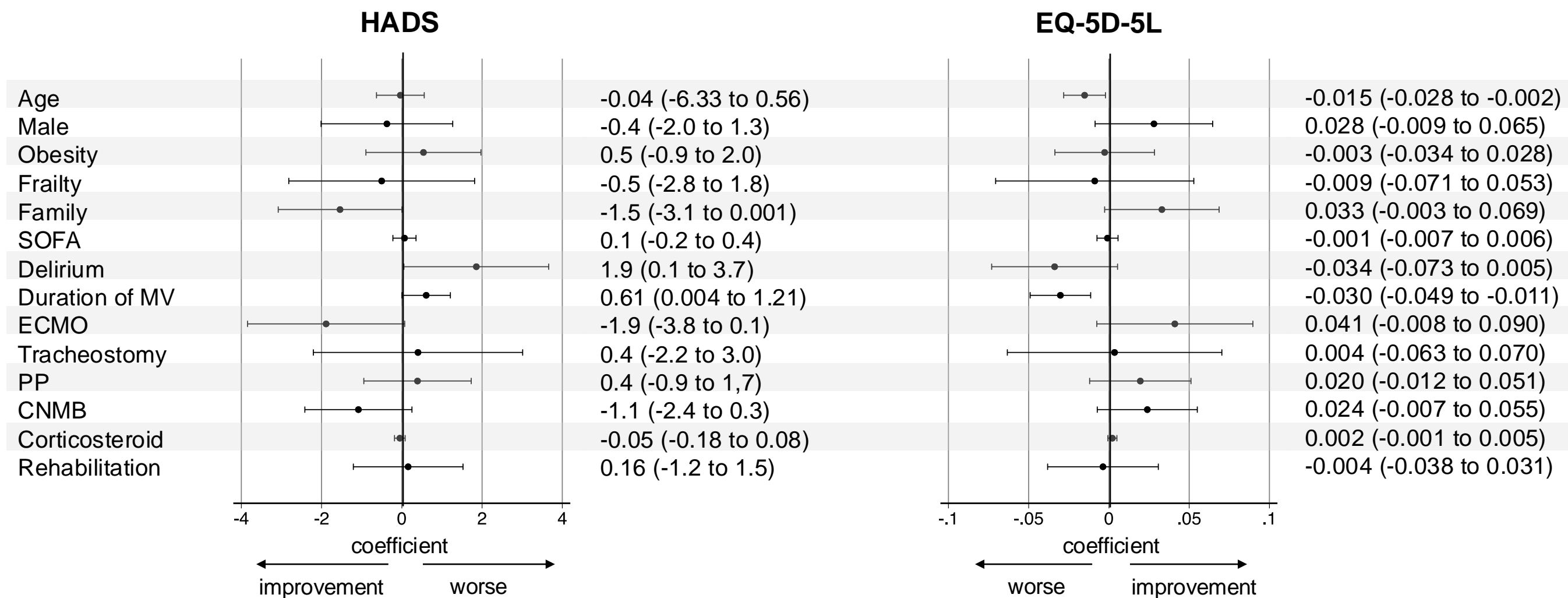

Supplement: Supplementary file 4 — Supplementary Material 4. [file 40560_2025_777_MOESM4_ESM.pdf]
