# Supplementary material for: Two-year trajectory of functional recovery and quality of life in post-intensive care syndrome: a multicenter prospective observational study on mechanically ventilated patients with coronavirus disease-19
Source: J Intensive Care. 2025 Feb 6;13:7. doi: 10.1186/s40560-025-00777-z (PMC11800417; doi:10.1186/s40560-025-00777-z)
Supplement: Supplementary file 5 — Supplementary Material 5. [file 40560_2025_777_MOESM5_ESM.docx]

**Supplemental Material**

**Two-year trajectory of functional recovery and quality of life in post-intensive care syndrome: A multicenter prospective observational study on mechanically ventilated patients with coronavirus disease-19**

Supplemental Table 1. Patient characteristics and clinical course in all questionnaires completed (n = 178)

Supplemental Table 2. Patient background of dropout patients before the first, second, third, and fourth surveys

Supplemental Table 3. Number of missing values from the first, second, third, and fourth patient survey results

Supplemental Table 4. Questionnaire results for all questionnaires completed (n = 178)

Supplemental Table 5. Patient characteristics and clinical course with and without extracorporeal membrane oxygenation

Supplemental Table 1. Patient characteristics and clinical course in all questionnaires completed (n = 178)

|  | n = 178 |
| --- | --- |
| Age, years, median (IQR) | 67 (58, 73) |
| Male, n (%) | 141 (79.2) |
| BMI, kg/m^2^, median (IQR) | 25.0 (22.8, 28.3) |
| Obesity (BMI ≥25), n (%) | 89 (50.0) |
| Living with family, n (%) | 144 (80.9) |
| SOFA score on the day of ventilation start, median (IQR) | 5 (4, 7) |
| PaO_2_/F_I_O_2_ ratio before mechanical ventilation | 133 (96, 185) |
| Clinical frailty scale | 2 (1, 2) |
| Frailty (Clinical frailty scale ≥4), n (%) | 9 (5.1) |
| ICU mobility scale |  |
| 3 days | 0 (0, 0) |
| 5 days | 0 (0, 1) |
| 7 days | 0 (0, 1) |
| Delirium, n (%) | 27 (15.2) |
| Duration of delirium within 1 week of ICU admission, day, median (IQR) | 2 (1, 4) |
| Duration of mechanical ventilation, day, median (IQR) | 9 (6, 15) |
| Length of ICU stay, day, median (IQR) | 11 (8, 18) |
| Length of hospital stay, day, median (IQR) | 21 (12, 38) |
| Comorbidity, n (%) | 121 (68.0) |
| hypertension | 83 (46.6) |
| diabetes mellitus | 53 (29.8) |
| cardiac disease | 16 (9.0) |
| COPD | 15 (8.4) |
| CKD G5 | 5 (2.8) |
| autoimmune disorder | 6 (3.4) |
| immunodeficiency | 4 (2.3) |
| malignant tumor | 10 (5.6) |
| Reintubation, n (%) | 8 (4.5) |
| Tracheostomy, n (%) | 35 (20.0) |
| ECMO, n (%) | 22 (12.4) |
| Duration of ECMO, day, median (IQR) | 10.5 (9, 18) |
| Prone position, n (%) | 99 (55.6) |
| Time from ICU admission to the prone position, day, median (IQR) | 1 (1, 3) |
| Duration of the prone position, day, median (IQR) | 4 (2, 5) |
| Prone position time per session, hour, median (IQR) | 16 (8, 17) |
| Continuous neuromuscular blocking agent, n (%) | 79 (44.4) |
| Duration of continuous neuromuscular blocking agent, day, median (IQR) | 2 (2, 3) |
| Corticosteroid, n (%) | 141 (79.2) |
| Maximum prednisolone dose, mg/day, median (IQR) | 42.7 (30, 100) |
| RRT, n (%) | 18 (10.1) |
| IRRT | 10 (5.6) |
| CRRT | 16 (9.0) |
| Rehabilitation program, n (%) | 97 (54.5) |
| Time from ICU admission to rehabilitation program initiation, day, median (IQR) | 5 (2, 16) |
| ICU diary, n (%) | 19 (10.7) |

BMI: body mass index, CKD: chronic kidney disease, COPD: chronic obstructive pulmonary disease, CRRT: continuous renal replacement therapy, ECMO: extracorporeal membrane oxygenation, ICU: intensive care unit, IQR: interquartile range, IRRT: intermittent RRT, RRT: renal replacement therapy, SOFA: sequential organ failure assessment

Supplemental Table 2. Patient background of dropout patients before the first, second, third, and fourth PICS surveys

The following table shows the patient background of each of the 83 patients who dropped out of the first survey from the enrolled patients, 42 patients who dropped out by the second survey, 17 patients who dropped out by the third survey, and 14 patients who dropped out by the fourth survey.

|  | First survey  n = 83 | Second survey  n = 42 | Third survey  n = 17 | Forth survey  n = 14 |
| --- | --- | --- | --- | --- |
| Age, years, median (IQR) | 68 (57, 73) | 66.5 (58, 75) | 70 (60, 75) | 69.5 (49, 76) |
| Male, n (%) | 66 (79.5) | 33 (78.6) | 15 (88.2) | 11 (78.6) |
| BMI, kg/m^2^, median (IQR) | 25.5 (23.1, 28.7) | 26.9 (22.7, 29.7) | 25.7 (23.4, 27.8) | 22.0 (18.6, 27.4) |
| Obesity (BMI ≥25), n (%) | 45 (54.2) | 26 (61.9) | 10 (58.8) | 5 (35.7) |
| Living with family, n (%) | 57 (68.7) | 25 (59.5) | 14 (82.4) | 10 (71.4) |
| SOFA score on the day of ventilation start, median (IQR) | 6 (4, 7) | 5.5 (4, 7) | 5 (3, 8) | 4 (4, 6) |
| PaO_2_/F_I_O_2_ ratio before mechanical ventilation | 129 (91, 178) | 110 (70, 189) | 129 (90, 163) | 133 (85, 205) |
| Clinical frailty scale | 2 (1, 3) | 2 (1, 3) | 1 (1, 2) | 2 (1, 3) |
| Frailty (Clinical frailty scale ≥4), n (%) | 6 (7.2) | 4 (9.5) | 0 (0) | 2 (14.3) |
| ICU mobility scale |  |  |  |  |
| 3 days | 0 (0, 1) | 0 (0, 0) | 0 (0, 0) | 0 (0, 0) |
| 5 days | 0 (0, 1) | 0 (0, 1) | 0 (0, 0) | 0.5 (0, 1) |
| 7 days | 1 (0, 1) | 0 (0, 1) | 0 (0, 1) | 1 (0, 3) |
| Delirium, n (%) | 13 (15.7) | 12 (28.6) | 6 (36.3) | 5 (35.7) |
| Duration of delirium within 1 week of ICU admission, day, median (IQR) | 2 (1, 3) | 1.5 (1, 3.5) | 2.5 (1, 3) | 3 (2, 4) |
| Duration of mechanical ventilation, day, median (IQR) | 7 (5, 11) | 9 (6, 16) | 8 (6, 19) | 9.5 (7, 14) |
| Length of ICU stay, day, median (IQR) | 9 (6, 14) | 11 (8, 21) | 14 (7, 27) | 11.5 (8, 16) |
| Length of hospital stay, day, median (IQR) | 16 (9, 31) | 29 (13, 39) | 25 (15, 43) | 29.5 (13, 53) |
| Comorbidity, n (%) | 57 (68.7) | 30 (71.4) | 8 (47.1) | 9 (64.3) |
| hypertension | 32 (38.6) | 18 (42.9) | 7 (41.2) | 6 (42.9) |
| diabetes mellitus | 34 (41.0) | 19 (45.2) | 5 (29.4) | 3 (21.4) |
| cardiac disease | 11 (13.3) | 8 (19.1) | 2 (11.8) | 0 (0) |
| COPD | 9 (10.8) | 4 (9.5) | 0 (0) | 2 (14.3) |
| CKD G5 | 2 (2.4) | 1 (2.4) | 1 (5.9) | 0 (0) |
| autoimmune disorder | 3 (3.6) | 2 (4.8) | 0 (0) | 1 (7.1) |
| immunodeficiency | 4 (4.8) | 3 (7.1) | 0 (0) | 1 (7.1) |
| malignant tumor | 8 (9.6) | 4 (9.5) | 0 (0) | 0 (0) |
| Reintubation, n (%) | 2 (2.4) | 1 (2.4) | 0 (0) | 1 (7.1) |
| Tracheostomy, n (%) | 9 (10.8) | 12 (28.6) | 4 (23.5) | 0 (0) |
| ECMO, n (%) | 13 (15.7) | 8 (19.1) | 2 (11.8) | 1 (7.1) |
| Duration of ECMO, day, median (IQR) | 12 (9, 16) | 15 (8.5, 17) | 22 (12, 32) | 9 (9, 9) |
| Prone position, n (%) | 22 (26.5) | 19 (45.2) | 6 (35.3) | 8 (57.1) |
| Time from ICU admission to the prone position, day, median (IQR) | 1 (1, 2) | 1 (1, 1) | 1 (1, 7) | 1 (1, 4.5) |
| Duration of the prone position, day, median (IQR) | 4 (3, 6) | 4 (2, 7) | 2 (1, 2) | 2.5 (2, 5.5) |
| Prone position time per session, hour, median (IQR) | 15 (10, 17) | 16 (12, 16) | 15 (8, 15) | 14.5 (8, 16) |
| Continuous neuromuscular blocking agent, n (%) | 42 (50.6) | 19 (45.2) | 7 (41.2) | 7 (50.0) |
| Duration of continuous neuromuscular blocking agent, day, median (IQR) | 3 (2, 4) | 2 (2, 2) | 3 (2, 8) | 2 (2, 2) |
| Corticosteroid, n (%) | 65 (78.3) | 30 (71.4) | 14 (82.4) | 9 (64.3) |
| Maximum prednisolone dose, mg/day, median (IQR) | 41.3 (30, 75) | 44 (0, 75) | 66 (30, 82.5) | 36.5 (0, 75) |
| RRT, n (%) | 6 (7.2) | 2 (4.8) | 2 (11.8) | 1 (7.1) |
| IRRT | 3 (3.6) | 2 (4.8) | 1 (5.9) | 0 (0) |
| CRRT | 5 (6.0) | 1 (2.4) | 1 (5.9) | 1 (7.1) |
| Rehabilitation program, n (%) | 42 (50.6) | 26 (61.9) | 8 (47.1) | 9 (64.3) |
| Time from ICU admission to rehabilitation program initiation, day, median (IQR) | 5 (2, 11) | 4 (2, 9) | 15 (2, 21) | 3 (1, 10) |
| ICU diary, n (%) | 9 (10.8) | 5 (11.9) | 3 (17.7) | 0 (0) |

BMI: body mass index, CKD: chronic kidney disease, COPD: chronic obstructive pulmonary disease, CRRT: continuous renal replacement therapy, ECMO: extracorporeal membrane oxygenation, ICU: intensive care unit, IQR: interquartile range, IRRT: intermittent RRT, RRT: renal replacement therapy, SOFA: sequential organ failure assessment

Supplemental Table 3. Number of missing values from the first, second, third, and fourth patient survey results

| Questions | Number of missing values | | | |
| --- | --- | --- | --- | --- |
|  | First survey  (n = 251) | Second survey  (n = 209) | Third survey  (n = 192) | Forth survey  (n = 178) |
| Dyspnea, n(%) | 0 (0) | 3 (1.4) | 3 (1.6) | 0 (0) |
| Walking difficulty, n(%) | 2 (0.8) | 4 (1.9) | 3 (1.6) | 0 (0) |
| Weight loss, n(%) | 0 (0) | 3 (1.4) | 2 (1.0) | 1 (0.6) |
| Memory impairment, n(%) | 2 (0.8) | 1 (0.5) | 2 (1.0) | 1 (0.6) |
| Execution disability, n(%) | 0 (0) | 4 (1.9) | 1 (0.5) | 2 (1.1) |
| Depression, n(%) | 0 (0) | 2 (1.0) | 1 (0.5) | 0 (0) |
| Anxiety, n(%) | 0 (0) | 1 (0.5) | 1 (0.5) | 0 (0) |
| Sleeping disorder, n(%) | 0 (0) | 1 (0.5) | 2 (1.0) | 0 (0) |
| Visual analogue scale, n(%) |  |  |  |  |
| Physical condition (on a scale of 1 to 10) | 1 (0.4) | 1 (0.5) | 1 (0.5) | 0 (0) |
| Cognitive function (on a scale of 1 to 10) | 2 (0.8) | 1 (0.5) | 1 (0.5) | 0 (0) |
| Mental health (on a scale of 1 to 10) | 1 (0.4) | 1 (0.5) | 1 (0.5) | 0 (0) |
| Barthel Index, n(%) | 4 (1.6) | 3 (1.4) | 1 (0.5) | 2 (1.1) |
| Short-Memory Questionnaire, n(%) | 5 (2.0) | 3 (1.4) | 2 (1.0) | 4 (2.3) |
| HADS score, n(%) |  |  |  |  |
| HADS-Anxiety score | 1 (0.4) | 0 (0) | 1 (0.5) | 1 (0.6) |
| HADS-Depression score | 1 (0.4) | 0 (0) | 1 (0.5) | 1 (0.6) |
| EQ-5D-5L, n(%) | 4 (1.6) | 6 (2.9) | 6 (3.1) | 4 (2.3) |

EQ-5D-5L; EuroQol 5 dimensions 5-level, HADS; Hospital Anxiety and Depression Scale

Supplemental Table 4. Questionnaire results for all questionnaires completed (n = 178)

Outcome scores of Barthel index, Short-Memory Questionnaire, Hospital Anxiety and Depression Scale-Anxiety score, Hospital Anxiety and Depression Scale-Depression score, and EuroQol 5 dimensions 5-level were stabilized using inverse probability of censoring weights that take into account missing data. The included covariates were age, gender, BMI, SOFA score, clinical frailty scale, comorbidities (hypertension, diabetes, cardiac disease, chronic renal disease, autoimmune disease, malignancy, chronic obstructive pulmonary disease, immunodeficiency), reintubation, ECMO, tracheostomy, continuous neuromuscular blockade, supine position, maximum prednisolone equivalent daily dose, continuous renal replacement therapy, intermittent renal replacement therapy, rehabilitation, delirium, duration of mechanical ventilation, length of ICU stay, length of hospital stay, ICU duration, presence of family members, and use of an ICU diary.

|  | 1st survey 5.5 (3.1) months after ICU discharge | 2nd survey  12.5 (3.1) months after ICU discharge | 3rd survey  18.5 (3.1) months after ICU discharge | 4th survey  24.5 (3.1) months after ICU discharge |
| --- | --- | --- | --- | --- |
| **Physical function** |  |  |  |  |
| Barthel index, mean (SD) | 92.3 (29.9) | 95.3 (47.3) | 94.2 (37.2) | 94.4 (37.1) |
| Self-care impairment, n (%) | 22 (12.4) | 22 (12.4) | 14 (7.9) | 16 (9.0) |
| Excretion impairment, n (%) | 23 (12.9) | 19 (10.7) | 25 (14.0) | 23 (12.9) |
| Transferring impairment, n (%) | 9 (5.1) | 7 (3.9) | 8 (4.5) | 8 (4.5) |
| Movement impairment, n (%) | 23 (12.9) | 27 (15.2) | 18 (10.1) | 22 (12.4) |
| **Cognitive function** |  |  |  |  |
| Short-Memory Questionnaire, mean (SD) | 37.7 (12.6) | 38.4 (21.3) | 36.6 (15.4) | 36.9 (15.7) |
| **Mental health** |  |  |  |  |
| Anxiety (HADS-Anxiety score ≥8), n (%) | 32 (18.0) | 21 (11.8) | 29 (16.3) | 25 (14.0) |
| Depression (HADS-Depression score ≥8), n (%) | 37 (20.8) | 31 (17.4) | 32 (18.0) | 36 (20.2) |
| HADS score, mean (SD) | 9.1 (8.8) | 9.1 (12.7) | 9.1 (9.7) | 8.8 (8.0) |
| HADS-Anxiety score | 4.3 (4.4) | 4.3 (6.1) | 4.4 (5.1) | 3.8 (3.9) |
| HADS-Depression score | 4.8 (4.9) | 4.9 (7.0) | 4.8 (5.1) | 4.9 (4.8) |
| **QOL** |  |  |  |  |
| EQ-5D-5L, mean (SD) | 0.804 (0.295) | 0.846 (0.424) | 0.846 (0.359) | 0.824 (0.359) |
| **Visual analog scale, mean (SD)** |  |  |  |  |
| Physical condition (on a scale of 1 to 10) | 7.0 (2.0) | 7.2 (1.9) | 7.3 (1.9) | 7.1 (1.9) |
| Cognitive function (on a scale of 1 to 10) | 8.2 (1.9) | 8.1 (1.8) | 8.0 (1.9) | 8.0 (1.8) |
| Mental health (on a scale of 1 to 10) | 7.7 (2.2) | 7.6 (2.2) | 7.8 (2.2) | 7.8 (1.9) |
| **Others, n (%)** |  |  |  |  |
| Dyspnea | 90 (50.6) | 79 (44.4) | 81 (45.5) | 89 (50.0) |
| Walking difficulty | 59 (33.2) | 42 (23.6) | 48 (27.0) | 57 (32.0) |
| Weight loss | 112 (62.9) | 41 (23.0) | 36 (20.2) | 48 (27.0) |
| Memory impairment | 50 (28.1) | 57 (32.0) | 59 (33.2) | 68 (38.2) |
| Executive dysfunction | 87 (48.9) | 77 (43.3) | 87 (48.9) | 82 (46.1) |
| Depression | 72 (40.5) | 70 (39.3) | 61 (34.3) | 70 (39.3) |
| Anxiety | 101 (56.7) | 88 (49.4) | 84 (47.2) | 93 (52.3) |
| Sleeping disorder | 77 (43.3) | 79 (44.4) | 76 (42.7) | 73 (41.0) |

EQ-5D-5L: EuroQol 5 dimensions 5-level, HADS: Hospital Anxiety and Depression Scale, QOL: quality of life, SD: standard deviation

Supplemental Table 5. Patient characteristics and clinical course with and without extracorporeal membrane oxygenation

|  | ECMO  n = 46 | Non-ECMO  n = 288 | P value |
| --- | --- | --- | --- |
| Age, years, median (IQR) | 57.5 (48, 66) | 68 (60, 75) | <0.0001 |
| Male, n (%) | 41 (89.1) | 225 (78.1) | 0.09 |
| BMI, kg/m^2^, median (IQR) | 27.9 (25.6, 29.5) | 24.9 (22.3, 28.3) | <0.0001 |
| Obesity (BMI ≥25), n (%) | 36 (78.3) | 139 (48.3) | <0.0001 |
| Living with family, n (%) | 34 (73.9) | 216 (75.0) | 0.88 |
| SOFA score on the day of ventilation start, median (IQR) | 6 (4, 8) | 5 (4, 7) | 0.01 |
| PaO_2_/F_I_O_2_ ratio before mechanical ventilation | 97 (71, 136) | 135 (94, 190) | 0.0002 |
| Clinical frailty scale | 1 (1, 2) | 2 (1, 3) | 0.03 |
| Frailty (Clinical frailty scale ≥4), n (%) | 0 (0) | 21 (7.3) | 0.06 |
| ICU mobility scale |  |  |  |
| 3 days | 0 (0, 0) | 0 (0, 0) | 0.36 |
| 5 days | 0 (0, 0) | 0 (0, 1) | 0.0002 |
| 7 days | 0 (0, 0) | 1 (0, 1) | <0.0001 |
| Delirium, n (%) | 9 (19.6) | 54 (18.8) | 0.90 |
| Duration of delirium within 1 week of ICU admission, day, median (IQR) | 1 (1, 2) | 2 (1, 4) | 0.20 |
| Duration of mechanical ventilation, day, median (IQR) | 19.5 (14, 30) | 7.5 (6, 12) | <0.0001 |
| Length of ICU stay, day, median (IQR) | 20 (15, 36) | 10 (7, 15) | <0.0001 |
| Length of hospital stay, day, median (IQR) | 38.5 (22, 55) | 18.5 (11, 35) | <0.0001 |
| Comorbidity, n (%) | 30 (65.2) | 195 (67.7) | 0.74 |
| hypertension | 16 (34.8) | 130 (45.1) | 0.19 |
| diabetes mellitus | 20 (43.5) | 94 (32.6) | 0.15 |
| cardiac disease | 3 (6.5) | 34 (11.8) | 0.29 |
| COPD | 3 (6.5) | 27 (9.4) | 0.53 |
| CKD G5 | 0 (0) | 9 (3.1) | 0.22 |
| autoimmune disorder | 2 (4.4) | 10 (3.5) | 0.77 |
| immunodeficiency | 1 (2.2) | 11 (3.8) | 0.58 |
| malignant tumor | 0 (0) | 22 (7.6) | 0.052 |
| Reintubation, n (%) | 0 (0) | 12 (4.2) | 0.16 |
| Tracheostomy, n (%) | 20 (43.5) | 40 (13.9) | <0.0001 |
| Prone position, n (%) | 20 (43.5) | 134 (46.5) | 0.70 |
| Time from ICU admission to the prone position, day, median (IQR) | 4 (1.5, 8.5) | 1 (1, 2) | <0.0001 |
| Duration of the prone position, day, median (IQR) | 5 (2, 7) | 3 (2, 5) | 0.19 |
| Prone position time per session, hour, median (IQR) | 12 (8, 16) | 16 (10, 17) | 0.047 |
| Continuous neuromuscular blocking agent, n (%) | 31 (67.4) | 123 (42.7) | 0.002 |
| Duration of continuous neuromuscular blocking agent, day, median (IQR) | 2 (2, 5) | 2 (2, 3) | 0.14 |
| Corticosteroid, n (%) | 22 (47.8) | 237 (82.3) | <0.0001 |
| Maximum prednisolone dose, mg/day, median (IQR) | 0 (0, 50) | 44 (30, 100) | 0.0003 |
| RRT, n (%) | 9 (19.6) | 20 (6.9) | 0.005 |
| IRRT | 4 (8.7) | 12 (4.2) | 0.18 |
| CRRT | 7 (15.2) | 17 (5.9) | 0.02 |
| Rehabilitation program, n (%) | 30 (65.2) | 152 (52.8) | 0.12 |
| Time from ICU admission to rehabilitation program initiation, day, median (IQR) | 5 (3, 17) | 5 (2, 15) | 0.34 |
| ICU diary, n (%) | 7 (15.2) | 29 (10.1) | 0.30 |

BMI: body mass index, CKD: chronic kidney disease, COPD: chronic obstructive pulmonary disease, CRRT: continuous renal replacement therapy, ECMO: extracorporeal membrane oxygenation, ICU: intensive care unit, IQR: interquartile range, IRRT: intermittent RRT, RRT: renal replacement therapy, SOFA: sequential organ failure assessment
